# Type I Collagen as an Extracellular Matrix for the *In Vitro* Growth of Human Small Intestinal Epithelium

**DOI:** 10.1371/journal.pone.0107814

**Published:** 2014-09-15

**Authors:** Ziyad Jabaji, Garrett J. Brinkley, Hassan A. Khalil, Connie M. Sears, Nan Ye Lei, Michael Lewis, Matthias Stelzner, Martín G. Martín, James C. Y. Dunn

**Affiliations:** 1 Department of Surgery, Division of Pediatric Surgery, David Geffen School of Medicine at UCLA, University of California Los Angeles, Los Angeles, California, United States of America; 2 Department of Bioengineering, Henry Samueli School of Engineering, University of California Los Angeles, Los Angeles, California, United States of America; 3 Department of Pathology, Veterans Affairs Greater Los Angeles Healthcare System, Los Angeles, California, United States of America; 4 Department of Pediatrics, Division of Gastroenterology and Nutrition, Mattel Children's Hospital and the David Geffen School of Medicine at UCLA, University of California Los Angeles, Los Angeles, California, United States of America; 5 Department of Surgery, Veterans Affairs Greater Los Angeles Healthcare System, Los Angeles, California, United States of America; 6 Eli and Edythe Broad Center of Regenerative Medicine & Stem Cell Research, University of California Los Angeles, Los Angeles, California, United States of America; Rush University Medical Center, United States of America

## Abstract

**Background:**

We previously reported *in vitro* maintenance and proliferation of human small intestinal epithelium using Matrigel, a proprietary basement membrane product. There are concerns over the applicability of Matrigel-based methods for future human therapies. We investigated type I collagen as an alternative for the culture of human intestinal epithelial cells.

**Methods:**

Human small intestine was procured from fresh surgical pathology specimens. Small intestinal crypts were isolated using EDTA chelation. Intestinal subepithelial myofibroblasts were isolated from a pediatric sample and expanded *in vitro*. After suspension in Matrigel or type I collagen gel, crypts were co-cultured above a confluent layer of myofibroblasts. Crypts were also grown in monoculture with exposure to myofibroblast conditioned media; these were subsequently sub-cultured *in vitro* and expanded with a 1∶2 split ratio. Cultures were assessed with light microscopy, RT-PCR, histology, and immunohistochemistry.

**Results:**

Collagen supported viable human epithelium *in vitro* for at least one month in primary culture. Sub-cultured epithelium expanded through 12 passages over 60 days. Histologic sections revealed polarized columnar cells, with apical brush borders and basolaterally located nuclei. Collagen-based cultures gave rise to monolayer epithelial sheets at the gel-liquid interface, which were not observed with Matrigel. Immunohistochemical staining identified markers of differentiated intestinal epithelium and myofibroblasts. RT-PCR demonstrated expression of α-smooth muscle actin and vimentin in myofibroblasts and E-Cadherin, *CDX2*, villin 1, intestinal alkaline phosphatase, chromogranin A, lysozyme, and Lgr5 in epithelial cells. These markers were maintained through several passages.

**Conclusion:**

Type I collagen gel supports long-term *in vitro* maintenance and expansion of fully elaborated human intestinal epithelium. Collagen-based methods yield familiar enteroid structures as well as a new pattern of sheet-like growth, and they eliminate the need for Matrigel for *in vitro* human intestinal epithelial growth. Future research is required to further develop this cell culture system for tissue engineering applications.

## Introduction

The last decade of research into the intestinal epithelial stem cell niche has been marked by exciting new insights which have resulted in numerous experimental systems of long-term *in vitro* epithelial growth. This work has been facilitated by the identification of critical growth factors, the use of three-dimensional culture techniques that provide a near-physiologic extracellular environment, and better characterization of supportive cells within the niche [1]–[4]. Though these culture systems have proven essential for advancement of *in vitro* research in the field, few studies have sought to further develop their clinical applicability. In particular, Matrigel, the most widely used extracellular matrix in epithelial cell culture, is unlikely to make the transition from bench to bedside as it is a poorly characterized mouse sarcoma digestate [5]. Furthermore, its inherent batch-to-batch variability is a significant obstacle in planning reproducible *in vitro* studies [6].

We have previously investigated type I collagen as a clinically and scientifically relevant alternative [7]. Collagen is the most abundant protein in animals, with twenty-eight distinct subtypes identified to date [8]. Type I collagen, the predominant subtype, is found mostly in connective tissue; can be easily and reproducibly isolated; and is relatively non-immunogenic. Importantly, there are several precedents for the use of collagen as a biomaterial in the clinical setting [9]–[12]. Given these clear advantages over existing three-dimensional culture systems, its application in intestinal regeneration is a promising avenue to direct current investigation.

In this investigation, we further develop three-dimensional human small intestinal epithelial culture methodology by demonstrating the capacity of type I collagen gel to replace Matrigel. By synthesizing insights from the collagen-based murine small intestinal model as well as prior techniques for human small intestinal culture, we propose a new direction for clinically-relevant approaches to *in vitro* intestinal epithelial culture.

## Materials and Methods

### Ethics Statement

All human tissues used in this study were obtained from discarded surgical specimens following clinical surgical pathology evaluation. This was approved by the UCLA Institutional Review Board (IRB #11-002189). The IRB waived the requirement for informed consent for tissues obtained from the UCLA Translational Pathology Core Laboratory (IRB #11-002504).

### Intestinal Subepithelial Myofibroblast Isolation, Culture, and Characterization

Intestinal subepithelial myofibroblasts (ISEMFs) were isolated from an enterectomy performed on an infant. The jejunal sample was washed vigorously with phosphate buffered saline (PBS) (Fisher Scientific, Pittsburgh, PA), and the mucosa was separated from the seromuscular layer using sharp dissection. The epithelium was removed from the mucosa according to a previously described protocol [13], and the mucosal remnant was incubated for 3 weeks in ISEMF medium consisting of Dulbecco's modified Eagle medium (DMEM)/Low Glucose/GlutaMAX (Invitrogen, Carlsbad, CA), 10% fetal bovine serum (FBS) (Invitrogen), 1x Antibiotic-Antimycotic (Invitrogen), 0.25 units/mL insulin (Sigma, St. Louis, MO), 20 ng/mL recombinant murine EGF (PeproTech, Rocky Hill, NJ), and 10 µg/mL transferrin (Sigma).

After they migrated from the mucosal remnants, the ISEMF population was sub-cultured and expanded as follows. When the ISEMFs reached confluency, they were gently washed with sterile PBS, treated with 1x TrypLE (Invitrogen), resuspended in ISEMF medium, and re-plated at a concentration of 200,000 ISEMFs/75 cm^2^. After iterative expansion through the first four passages *in vitro*, the ISEMFs were frozen in liquid nitrogen in 10% dimethyl sulfoxide (ATCC, Manassas, VA), 40% FBS, and 50% DMEM as a cryoprotectant for future studies. The ISEMFs were subsequently characterized by immunofluorescent staining and messenger RNA assessment as described below.

### Small Intestinal Crypt Isolation

Human small intestinal tissue samples were procured from pancreatico-duodenectomy, enterectomy, and ileostomy reversal surgical pathology specimens. The seromuscular layer was sharply dissected off of the overlying mucosa and discarded. Crypts were isolated from the mucosa as previously described with slight modification [4]; 8 mM ethylenediaminetetraacetate (EDTA) (Sigma) and 1 mM dithiothreitol (DTT) (Sigma) were used for the dissociation step, and crypts were only filtered through a 100 µm pore cell strainer (BD Biosciences, Bedford MA). Crypts were resuspended in 5 mL of Basic Crypt Medium, consisting of Advanced DMEM/Ham's F12 (Invitrogen) with 2 mM GlutaMAX (Invitrogen), 10 mM HEPES (Invitrogen), and 1x Antibiotic-Antimycotic (Invitrogen). Each experiment performed represented a distinct patient sample, and specimens were not pooled.

### Crypt Culture Technique

ISEMFs were pre-plated at an initial density of 25,000 cells/cm^2^ within 48-well Costar cell culture plates (Fisher) and allowed to grow to confluency several days before crypt isolation. Standard formulation Matrigel (BD Biosciences) was thawed on ice, and a type I collagen gel (Cellmatrix type I-A porcine tendon collagen, 3 mg/mL, Nitta Gelatin Inc., Osaka, Japan) was also prepared as previously described [7]. Crypts were counted and suspended within Matrigel or collagen, at a concentration of 250 crypts per 25 µL of either gel; the three-dimensional suspension was then plated over the confluent monolayer of ISEMFs. Cultures were treated with 250 µL of Complete Crypt Medium (CCM) consisting of Basic Crypt Medium, 1 mM N-Acetylcysteine (Sigma), 100 ng/mL recombinant murine Noggin (PeproTech), 50 ng/mL recombinant murine EGF (PeproTech), 1x N2 supplement (Invitrogen), 1x B27 supplement (Invitrogen), 1 µg/mL recombinant human (rh) R-spondin 1 (R&D Systems, Minneapolis, MN), 100 ng/mL of rhWnt3A (R&D Systems), and 100 ng/mL of rhFGF10 (Peprotech). Fresh CCM was replaced every 4 days, and growth factors (Noggin, EGF, R-spondin 1, Wnt3A, and FGF10) were supplemented 2 days after each new medium delivery.

Culture growth was assessed with inverted light microscopy on a Nikon Eclipse Ti-U (Nikon Instruments Inc., Melville, NY). A subset of culture wells were terminated after one week *in vitro* by overnight fixation with 10% buffered formalin with subsequent histologic analysis (see below) or lysis in RNA stabilization buffer.

### Intestinal Epithelial Sub-culture Technique

For intestinal epithelial sub-culturing, several experiments were initiated with crypt monocultures in the presence of ISEMF-conditioned media. ISEMF-conditioned Complete Crypt Medium (IC-CCM) was prepared in the following manner. ISEMFs were sub-cultured into a T-75 culture flask (Corning, Tewksbury, MA) and treated with ISEMF medium. The media was removed after one week of incubation and stored in a 4°C refrigerator for subsequent use. IC-CCM was constituted prior to each use by preparing a 1∶1 mixture of the spent ISEMF medium with doubly-concentrated CCM, such that the final concentration of the specific nutrients and growth factors above was unchanged. 2.5 µM CHIR99021 (Stemgent, Cambridge, MA) and 10 µM Y-27632 (Sigma) were also added.

Small intestinal crypts were isolated and suspended as above. They were then plated into 48-well Nunclon Delta-treated cell culture plates (Thermo Scientific, Waltham, MA) and treated with IC-CCM. The resultant epithelial units were sub-cultured every 4–5 days with a 1∶2 split ratio, using type XI-S collagenase (Sigma) and gentle mechanical disruption. They were then resuspended in collagen gel and re-plated followed by continued IC-CCM treatment.

### Immunofluorescent Staining and Histologic Analysis

Sub-cultured ISEMFs were characterized by *in situ* immunofluorescent staining. ISEMFs were fixed overnight in 10% buffered formalin, following by permeabilization with 0.5% Triton-X (Sigma) in Tris buffer solution and washing with 0.05% Tween 20 (EMD Chemicals, Philadelphia, PA) in PBS. Primary antibodies against α-smooth muscle actin (SMA) (Dako, Carpinteria, CA), vimentin (Abcam, Cambridge, MA), and desmin (Dako) were incubated overnight at 4°C at a 1∶50 dilution. After washing in PBS, AlexaFluor-488 conjugated secondary antibodies (Invitrogen) were incubated for 30 minutes at room temperature at a 1∶200 dilution, and nuclei were visualized with DAPI and Vectashield (Vector Labs, Burlingame, CA).

*In vitro* epithelial cultures were fixed overnight in 10% buffered formalin and embedded in paraffin blocks. Serial 5 µm sections were cut and prepared for either hematoxylin and eosin or immunohistochemical staining. Immunostaining was performed with antibodies directed against caudal type homeobox 2 (Cdx2), E-cadherin, CD10, lysozyme, chromogranin A, and α-smooth muscle actin. Mucin was detected using Periodic Acid-Schiff-Green staining [7].

### Polymerase Chain Reaction

Messenger RNA (mRNA) was isolated from sub-cultured ISEMFs and intestinal epithelial cultures with the RNeasy Mini Kit (Qiagen, Valencia, CA). Reverse transcriptase polymerase chain reaction (RT-PCR) was performed to determine the expression levels of genes of interest, using Taqman Gene Expression Assays (Applied Biosystems, Carlsbad, CA) for smooth muscle actin (*ACTA2*), vimentin (*VIM*), desmin (*DES*), E-Cadherin (*CDH1*), caudal type homeobox 2 (*CDX2*), villin 1 (*VIL1*), intestinal alkaline phosphatase (*ALPI*), chromogranin A (*CHGA*), lysozyme (*LYZ*), Lgr5 (*LGR5*), and glyceraldehyde-3-phosphate dehydrogenase (*GAPDH*), as previously described [7]. RT-PCR reactions were performed on a Prism 7900 HT Sequence Detection System (Applied Biosystems). Cycle numbers were analyzed according to the comparative C^T^ method using *GAPDH* as the internal calibrator and human whole small intestine as the reference tissue [14].

### Statistical Analysis

Two-tailed paired Student's t-test was used to compare results from the experiments involving primary culture. Single factor analysis of variance (ANOVA) was used to compare mRNA results from different passages from the sub-cultured experiments.

## Results

### ISEMF Characterization

**Figure 1:**
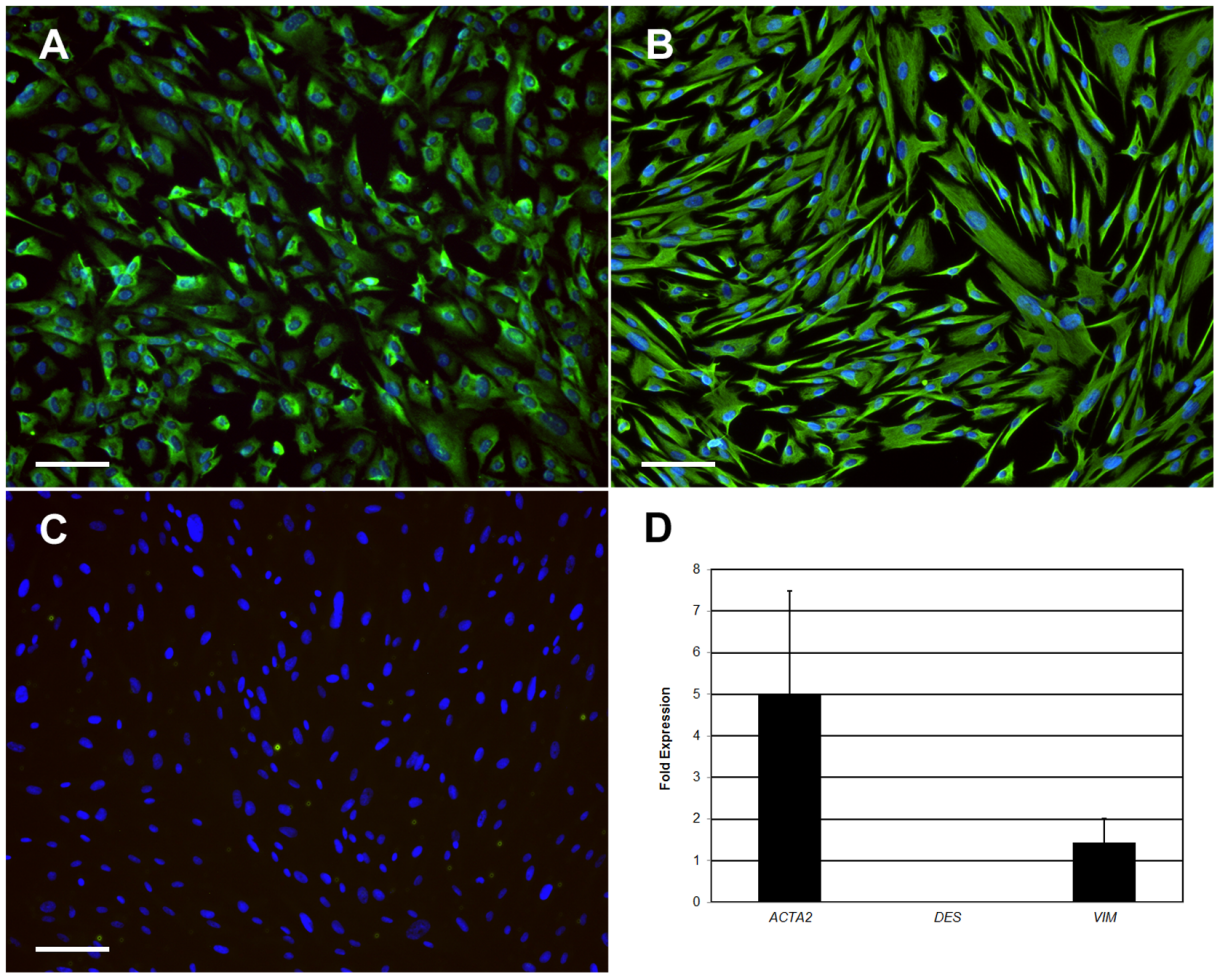
Characterization of intestinal subepithelial myofibroblast phenotype. **A–C:** Immunofluorescent staining against α-smooth muscle actin (**A**), vimentin (**B**), and desmin (**C**). Scale bar 100 µm (100x magnification). **D:** Gene expression by reverse transcriptase polymerase chain reaction. *ACTA2*: α-smooth muscle actin. *DES*: desmin. *VIM*: vimentin.

Sub-cultured ISEMFs appeared as elongated spindle-shaped cells *in vitro*. The characteristic phenotype of weak SMA expression, robust vimentin expression, and minimal desmin expression was confirmed by both immunofluorescence and RT-PCR (Figure 1). Thawed ISEMFs subsequently demonstrated the same characteristic immunophenotype (data not shown).

### *In Vitro* Intestinal Epithelial Growth in Primary Co-culture with ISEMFs

**Figure 2:**
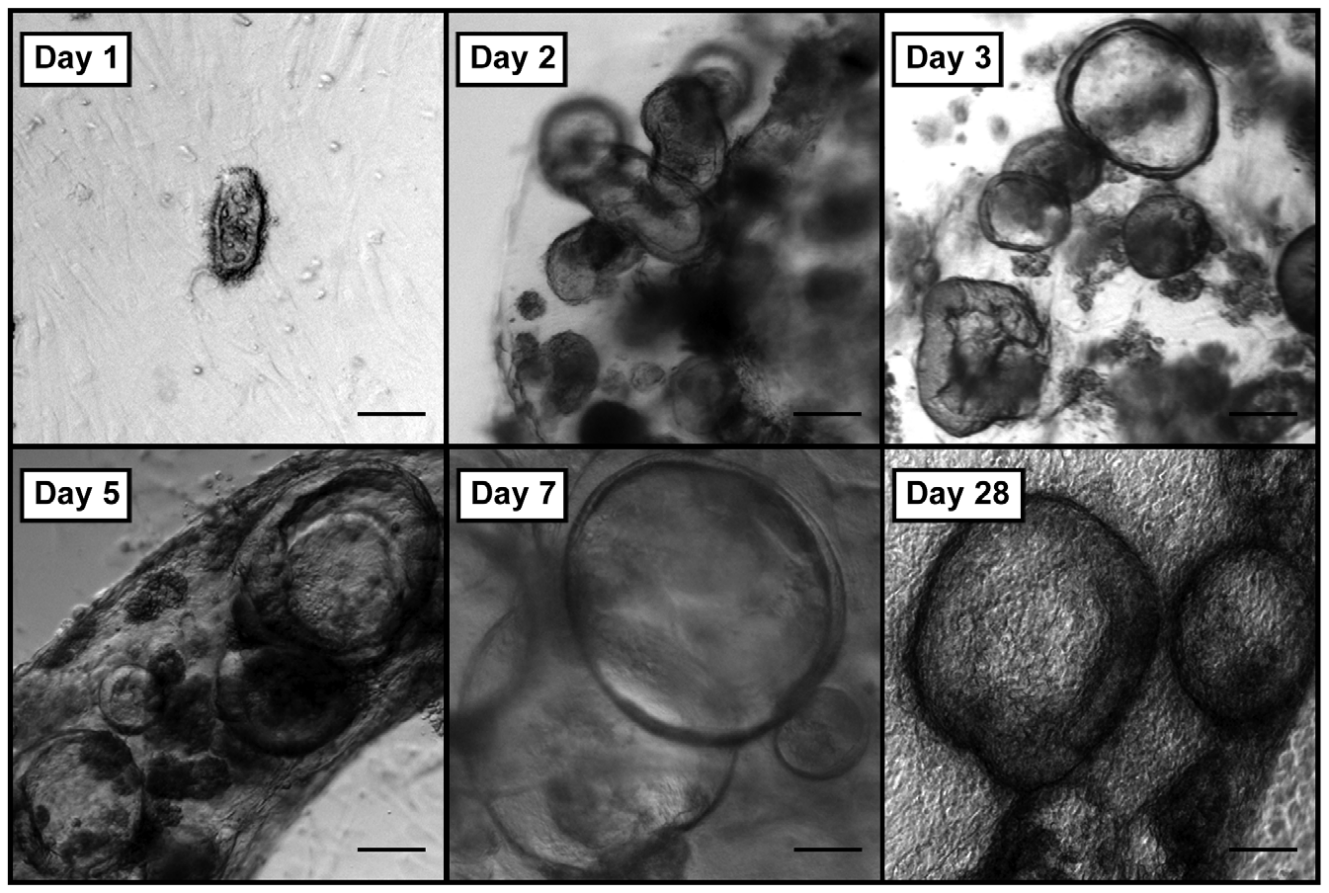
*In vitro* growth of human intestinal enteroids co-cultured with intestinal subepithelial myofibroblasts in collagen gel. Scale bar 100 µm (40x magnification).

**Figure 3:**
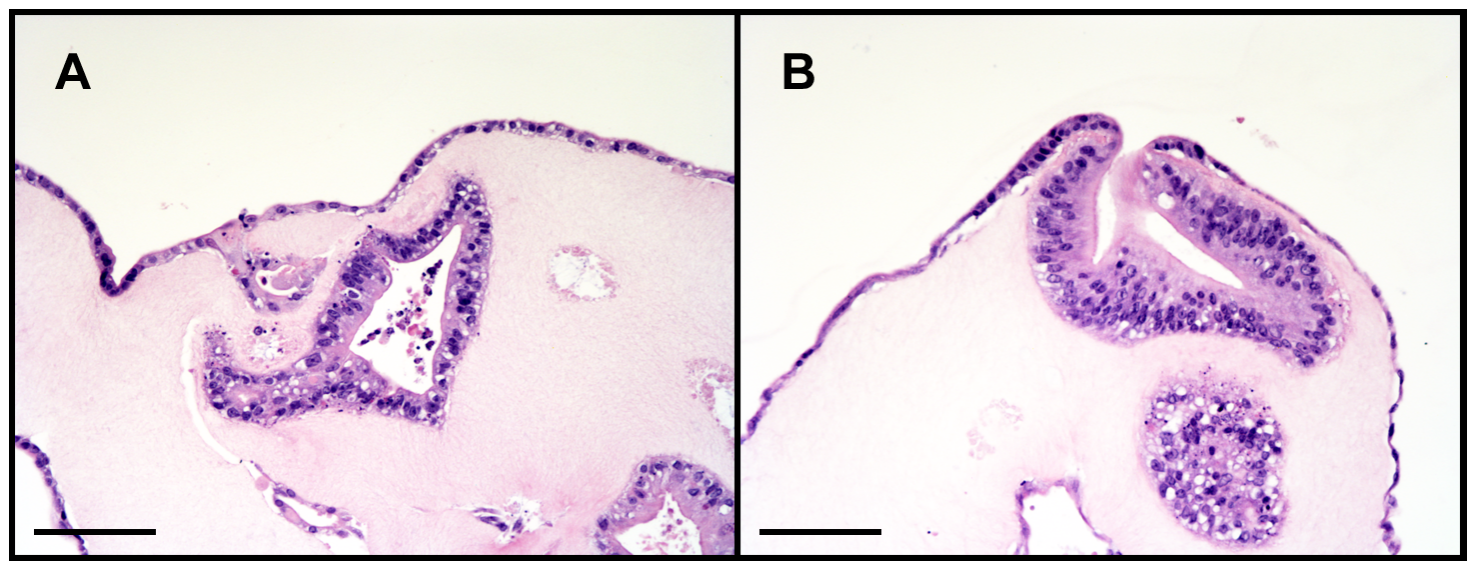
Hematoxylin and eosin stained sections of human small intestinal epithelium in collagen gel. Both enteroids and monolayer epithelial sheets are noted (**A**), with confluence of these two domains at areas where the enteroids have grown or migrated to the surface of the collagen gel (**B**). Scale bar 100 µm (200x magnification).

Human small intestinal crypts suspended in collagen gel consistently gave rise to *in vitro* epithelial units previously termed “enteroids” when grown in co-culture with ISEMFs (N = 8) [15]. Crypts plated in monoculture *without* IC-CCM underwent autolysis within 2–3 days. Co-cultured enteroids on top of ISEMFs rapidly grew over the first week after initial isolation (Figure 2). Thereafter, their growth slowed but remained viable with sharp basolateral borders for up to one month in primary culture (Figure 2, final panel). Moreover, cultured human epithelium demonstrated a tendency to form a monolayer of cells at the collagen-media interface (Figure 3). Crypts suspended in Matrigel similarly yielded enteroids only in the presence of ISEMF co-culture [4]. No surface epithelial sheets were noted to arise from Matrigel-based cultures.

### Assessment of Cell Lineage Differentiation

**Figure 4:**
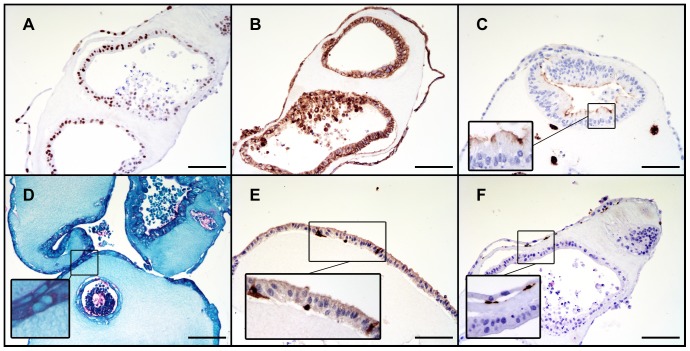
Immunohistochemical evidence of intestinal epithelial cell lineage differentiation in collagen. **A:** Cdx2 (caudal type homeobox 2). **B:** E-Cadherin. **C:** CD10. **D:** Periodic Acid-Schiff. **E:** Chromogranin A. **F:** α-smooth muscle actin. Scale bar 100 µm (200x magnification).

**Figure 5:**
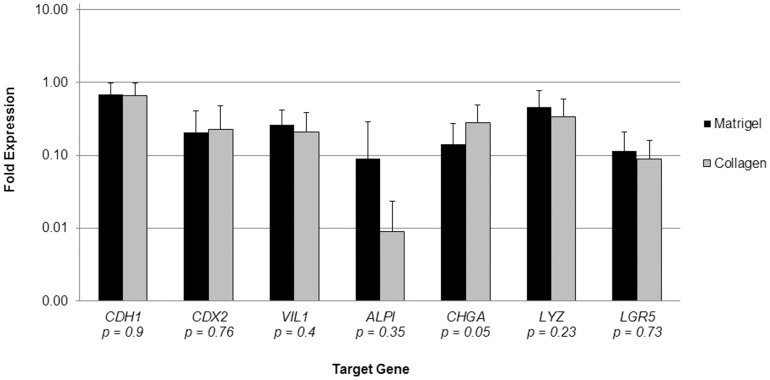
Gene expression profile of enteroids co-cultured with intestinal subepithelial myofibroblasts, relative to native small intestine, on a logarithmic scale. Reverse transcriptase polymerase chain reaction was performed on mRNA isolated from co-cultures after 1 week *in vitro*, using GAPDH as an internal calibrator gene. N = 7 for Matrigel, and N = 6 for collagen. Error bars denote standard deviation, and p-values are reported for Student's t-test comparing individual genes. *CDH1*: E-Cadherin. *CDX2*: caudal type homeobox 2. *VIL1*: villin 1. *ALPI*: intestinal alkaline phosphatase. *CHGA*: chromogranin A. *LYZ*: lysozyme. *LGR5*: leucine-rich repeat-containing G-protein coupled receptor 5.

The phenotypic differentiation of cultured epithelium was assessed immunohistochemically as well as by RT-PCR. Collagen-based cultures yielded staining patterns characteristic of differentiated intestinal epithelial cell lineages. They stained positively for absorptive enterocyte (Cdx2, E-Cadherin, CD10), goblet (Periodic Acid-Schiff), and enteroendocrine (Chromogranin A) cells; further, anti-SMA staining verified the expected subepithelial position of the supportive ISEMFs (Figure 4). The RT-PCR data confirmed these findings and in addition provided evidence of Paneth cell (*LYZ*) differentiation (Figure 5). Chromogranin A demonstrated the only statistically significant difference in mRNA expression levels between Matrigel- and collagen-based cultures among the target genes assessed.

### *In Vitro* Intestinal Epithelial Sub-culturing

**Figure 6:**
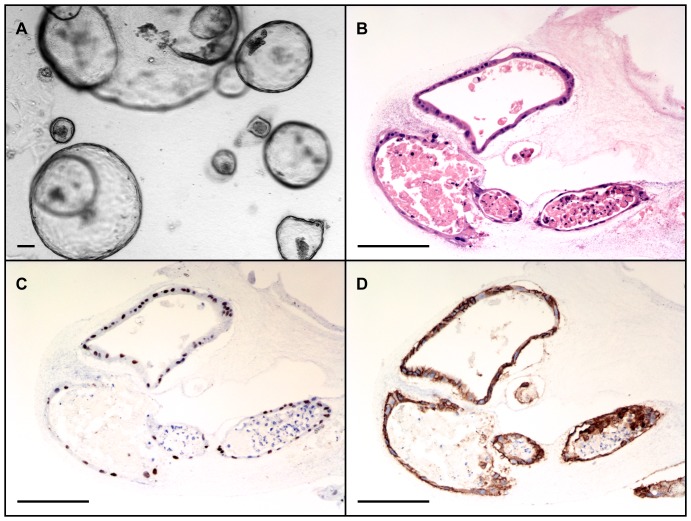
*In vitro* growth of human intestinal epithelium in monoculture with exposure to myofibroblast conditioned medium. **A:** Monocultured enterospheres appear as simpler, thinner-walled cysts. **B:** Hematoxylin and eosin-stained cross-section of an enterosphere. **C:** Cdx2 staining. **D:** E-Cadherin staining. Scale bar 100 µm (**A:** 40x magnification; **B–D:** 200x magnification).

**Figure 7:**
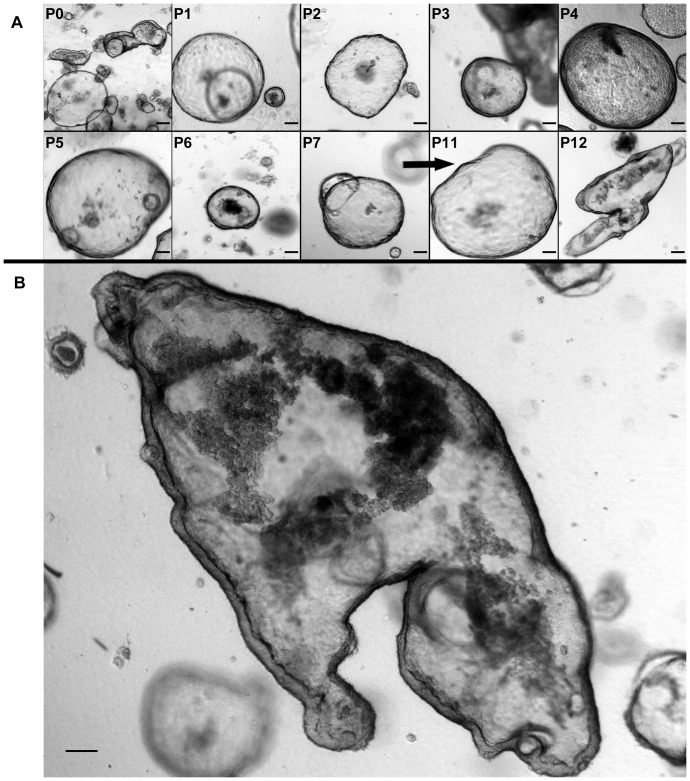
A: Sub-culturing of monocultured enterospheres with exposure to myofibroblast conditioned medium. ‘P’ denotes passage number; passages 8–10 are omitted for brevity. **B:** Large enteroid formed by fusion of passaged enterospheres. Scale bar 100 µm (40x magnification).

**Figure 8:**
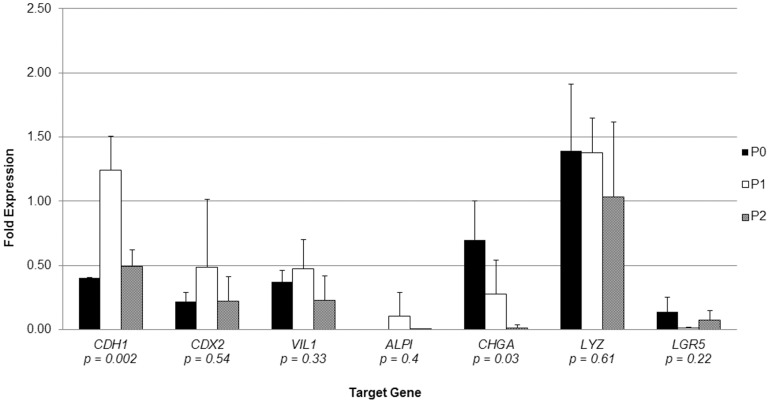
Gene expression profile of enterospheres monocultured in collagen gel, with exposure to myofibroblast conditioned medium. Reverse transcriptase polymerase chain reaction was performed on mRNA using GAPDH as an internal calibrator gene relative to native small intestine. ‘P’ denotes passage number; N = 3 for each passage. Error bars denote standard deviation, and p-values are reported for single factor analysis of variance (ANOVA) comparing individual genes across all passages. *CDH1*: E-Cadherin. *CDX2*: caudal type homeobox 2. *VIL1*: villin 1. *ALPI*: intestinal alkaline phosphatase. *CHGA*: chromogranin A. *LYZ*: lysozyme. *LGR5*: leucine-rich repeat-containing G-protein coupled receptor 5.

Small intestinal crypts reproducibly grew in collagen-based monoculture when exposed to IC-CCM (N = 3). In the absence of IC-CCM, crypts underwent autolysis in 2–3 days. Crypts grown in IC-CCM appeared as simpler cystic structures with flatter epithelialized walls (Figure 6), when compared with crypts co-cultured with ISEMFs (Figures 2,3). Hereafter we refer to these structures arising from IC-CCM supported monoculture as “enterospheres.” Enterospheres were successfully sub-cultured through 12 passages over the course of 60 days following initial crypt isolation (N = 1; Figure 7, panel A). Sub-cultured enterospheres retained the characteristic immunophenotype of intestinal epithelium (Figure 6, panels C/D). In addition, they were found to contain Chromogranin A and Lysozyme positive cells. RT-PCR demonstrated sustained expression of intestinal epithelial markers across several passages (Figure 8). On one occasion, passaged enterospheres appeared to have fused to form a very large enteroid (Figure 7, panel B), measuring approximately 2 mm by 1 mm in optical cross-section.

## Discussion

In this study, we have demonstrated that type I collagen can serve as a well-defined extra-cellular matrix for the reproducible *in vitro* growth of human small intestinal epithelium derived from healthy small intestinal crypts. The resultant cultures were shown to fully elaborate all major intestinal epithelial lineages. Enteroids co-cultured with ISEMFs were stable in collagen-based culture for one month in primary culture. Small intestinal enterospheres were successfully sub-cultured through 12 passages in the presence of ISEMF-conditioned medium. This work builds upon and highlights the key role that ISEMFs play in the *in vitro* intestinal epithelial stem cell niche [4], [16], [17].

The rapid proliferation in the last 5 years of experimental models for long-term *in vitro* gastrointestinal epithelial cell culture is a testament to an increasingly nuanced understanding of the intestinal stem cell niche, including its cellular players and cell-signaling mediators [18]. Since Sato's original description of long-term murine small intestinal epithelial culture [2], various reports have clarified the details necessary to culture the primary epithelia of human normal small intestine; normal and dysplastic colon; and metaplastic esophagus [4], [19], [20]. These studies have all employed Matrigel as an extracellular matrix for three-dimensional cell suspension. While its prolific application in mammalian cell culture spans over 2 decades [21], Matrigel ultimately remains a poorly defined and variable xenobiotic product derived from mouse sarcoma, with potential sterility concerns [5], [6], [22]. As such, the body of published work to date falls short of an ideal experimental model poised for translation into the clinic. These considerations have motivated efforts to supplant the use of Matrigel in other domains of stem cell research [23].

Similarly motivated by the goal of clinically translatable technology founded in good manufacturing practices, we and others have reported collagen-based culture systems for murine small intestinal epithelium [7], [24]. Matrigel-free systems have also been described for culturing immortalized human colorectal adenocarcinoma lines *in vitro* [25]–[28]. A recent report describes the *in vivo* generation of human small intestinal tissue derived from organoid units using biocompatible polymeric scaffolds and type I collagen gel coating [29]. However, this approach relies on the isolation of less cytologically pure stem cell-containing units; it also currently has the vexing disadvantage of requiring large amounts of starting material to generate relatively modest amount of tissue engineered small intestine.

To our knowledge, our system is unique as a biochemically- as well as cytologically-defined approach to the *in vitro* expansion and maintenance of non-transformed human small intestinal epithelium. This collagen-based system is advantageous for these reasons as well as for its capacity to expand a small amount of starting material *in vitro*, an important facet of intestinal tissue engineering paradigms. The Cellmatrix collagen product employed in this investigation has not yet been rigorously scrutinized as produced via good manufacturing practices. The future challenges notwithstanding, we anticipate its validation to be far less problematic than pursuing approval of Matrigel for clinical use, given the existing precendents of porcine collagen products already in clinical use [11], [30]. In contrast with Matrigel's well-described batch-to-batch variability [6], type I collagen has produced consistent results in our hands. This too will remain to be validated in a dedicated study in the future.

We are nonetheless faced with certain limitations to our current approach. Our system affords the capacity for long-term *in vitro* intestinal epithelial maintenance, but its support of enteroids or enterospheres *in vivo* has yet to be demonstrated. This will remain a critical task on the horizon. While the growth factor cocktail described by Sato and colleagues remains a cornerstone of recent progress in the field, we ultimately hope to achieve a fully autonomous system that recapitulates the intestinal stem cell niche *in vitro*. Early evidence utilizing ISEMFs suggests that our system may have the capacity to obviate the need for exogenous provision of growth factors, but further research is presently required to develop this potential. This observation of course begs the question of the mechanism of myofibroblast support of the intestinal stem cell niche, which we have not sought to further characterize in this study; this remains an area of active investigation [31].

In this investigation, we observed a new phenotype unique to the collagen-based system, the early appearance of an epithelial monolayer at the gel-liquid interface. Similar to prior experience in the murine model [7], epithelial sheets were ubiquitously seen in association with enteroids which had grown near the surface of the collagen (Figure 3). It is conceivable that this is merely a consequence of the natural behavior of polarized, basement membrane-anchored cells when geometric boundary conditions are imposed. However, it remains unclear why epithelial monolayers are observed in collagen-based cultures and yet are rare and late events when enteroids are grown in Matrigel. Despite the novel morphology of collagen-based cultures, the gene expression profiles of intestinal epithelium grown in Matrigel versus type I collagen gel were relatively similar (Figure 5). This may reflect a relative insensitivity of the selected target genes to characterize the obvious morphologic differences; whole transcriptome sequencing methods hold promise to better characterize the differences between the Matrigel and collagen phenotypes. In particular, we did observe a relatively low expression of intestinal alkaline phosphatase in both Matrigel- and collagen-based cultures (Figure 5); we attribute this to the “crypt-like” phenotype promoted by growth factor cocktail. The ability of gut epithelium to generate intestinal alkalkine phosphatase will be important in providing *in vivo* innate immunity [32], [33]. Our laboratory is performing ongoing experiments to induce further “villous-like” differentiation including up-regulation of this particular gene.

Further research should involve reconstitution of the other extracellular matrix constituents of Matrigel, as well as experimental manipulation of the collagen gel biomechanical properties [34]. The ability to leverage control over the specific intended morphology will be in an important tool in the future of intestinal tissue engineering. Ultimately, the capacity to grow organized epithelial sheets may aid in transitioning from micro-scale cultures to macro-scale neomucosa. The ability to promote formation of fused enteroid superstructures may also figure in future solutions to this challenge. This was an isolated and unexpected finding of our passaging methodology, and it will need to be validated and studied more extensively in the future.

The unique facets of collagen-based intestinal epithelial culture hold promise for future efforts in intestinal tissue engineering. Future effort will need to be directed towards understanding the mechanistic underpinning of the extracellular matrix-dependent phenomena described herein. Further development of this fully defined culture system will be important in supporting clinically-translatable technologies in the future.
